# Identifying complex patients in family medicine for potential benefit from a case manager: a short questionnaire derived from the INTERMED Self-Assessment (IMSA) questionnaire

**DOI:** 10.1186/s12875-022-01876-8

**Published:** 2022-11-04

**Authors:** Christine Cohidon, Emilie Gallay, Pascal Wild, Friedrich Stiefel, Céline Bourquin, Nicolas Senn

**Affiliations:** 1 Department of Family Medicine, Center for Primary Care and General Medicine (Unisanté), University of Lausanne, Lausanne, Switzerland; 2 Independent statistician, Nancy, France; 3 Psychiatric Liaison Service, Lausanne University Hospital and Lausanne University, Lausanne, Switzerland

**Keywords:** Case management, Intermed-SA tool, Complex patients, Family medicine

## Abstract

**Purpose:**

To investigate how useful the Intermed-Self Assessment (IMSA) questionnaire and its components were for identifying which patient candidates would benefit most from case management (CM) in general practice.

**Methods:**

The study was carried out in a group family medicine practice in Lausanne comprising seven GPs and four medical assistants, from February to April 2019. All the patients attending the practice between February and April 2019 were invited to complete the IMSA questionnaire. Additionally, their GPs were asked for their opinions on the potential benefits of each patient being assigned a case manager. Each IMSA item’s value has been assessed as a predictor of GPs’ opinions by using multivariate logistic models. A score including items retained as predictor was built.

**Results:**

Three hundred and thirty one patients participated in the study (participation rate: 62%). Three items from the 20 item IMSA were sufficient to predict GPs’ opinions about whether their patients could be expected to benefit if assigned a case manager. Those items addressed the patient’s existing chronic diseases (item1), quality of life in relation to existing diseases (item 3), and their social situation (item 9). Using these three items as a score, a cut-off at 4 gave a sensitivity of 70% (ability to correctly identify patients who could benefit from a CM) and specificity of 73% (ability to correctly identify patients who should not benefit from a CM) and concerned about one patient in two.

**Conclusion:**

Identifying complex patients suitable for case management remains a challenge for primary care professionals. This paper describes a novel approach using a structured process of combining the results of standardized tools such as the one defined in this study, and the experience of the primary care team.

**Supplementary Information:**

The online version contains supplementary material available at 10.1186/s12875-022-01876-8.

## Introduction

Faced with the challenges of an ageing population and increasing numbers of multi-morbid or complex patients, which create health and cost pressures, healthcare systems must aim to reduce the fragmented provision of care by promoting integrated care. In this context, many healthcare systems have introduced new models of care, often involving strengthening primary care and especially general practices [1–6]. Developing primary care teams [6–9] and introducing case managers [10–13] are two frequently chosen options. Indeed, case management has been recognized as an appropriate and highly responsive intervention to satisfy the particular needs of complex patients [14]. These patients, frequently managed in general practice, often suffer from combinations of multiple chronic conditions, mental health problems, drug interactions and social vulnerability [15]. In such context, case management generally allows to improve accessibility, quality and safety of care, continuity and coordination of care, leading to better, more efficient use of healthcare system resources and particularly a reduction in emergency room (ER) visits [13, 16–19].

However, identifying the candidates who would benefit most from case management is not easy. According to Garcia et al., complex patients are not always good candidates for intensive care management [20]. In addition, definitions of complexity in primary care are inconsistent, although they are often based on the frequency of use of healthcare services and the resulting costs [20]. Indeed, patients’ *choices* are often actually made by a professional—be they a physician, a nurse, or a case (care) manager—based on their clinical judgment. However, validated and standardized tools to measure the complexity, and at the same time, the interest of management by a case manager, offer an individual interest for the patient, but also collective interest for the health system. Both in hospitals and in primary care, it allows better anticipation of the patient’s trajectory in terms of future use of health services [21, 22]. In addition, such tools have been shown to be a good predictor of the patient quality of life, which is a key indicator to be considered in general medicine and primary care [23].

To the best of our knowledge, no specific tools exist to aid appropriate decision-making on whether a patient requires dedicated case management. Some tools do exist for measuring patient complexity as a predictor of future healthcare system use and, as mentioned above, frequent or inappropriate healthcare system use is currently one of the main reasons for needing a case manager. However, an additional purpose can be to screen for current clinical need and prognosis. One existing tool is the INTERMED Self-Assessment (IMSA) questionnaire [22], assessing patients’ biopsychosocial complexity and their past, present, and anticipated future health needs across four dimensions (physical, mental, social, and healthcare) [23–25]. To date, the IMSA questionnaire has rarely been used in primary care and general practice [26, 27]. In addition, regarding the objective of identifying the best patient candidates for case management, the IMSA questionnaire’s utility has yet to be demonstrated in Europe. In the USA, a modified version of the INTERMED is currently used in the teaching and practice of case management, but the approach is not specific to primary care and still needs to be evaluated [28]. The present study aimed to investigate how useful the IMSA questionnaire and its components were for identifying which patient candidates would benefit most from case management in general practice.

## Methods

### Setting and population

The present analysis was based on data from a preliminary study carried out to test the feasibility and acceptability of using the IMSA questionnaire in general practices. This was part of a larger pilot project (the MOCCA Project) planned by the Center for Primary Care and Public Health’s Department of Family Medicine and aimed at integrating nurses into general practices in the canton of Vaud (800’000 inhabitants), Switzerland [29]. In Switzerland, the current model for the provision of primary care via general practice (named family medicine) is a private sector activity. Approximatively half of the GPs work single-handedly, with limited or no multidisciplinary collaboration. They work almost exclusively with medical assistants, who support them in administrative tasks and by administering simple clinical procedures. Moreover, the demography of GPs is evolving towards a potential shortage [30]. The role of case (and care) manager is one of the most important foreseen for nurses in the MOCCA Project. The population targeted by this activity consisted of moderately complex patients—the goal being to prevent them from becoming highly complex patients. In Switzerland, highly complex patients with significant healthcare needs already benefit from home care services and care coordination provided in collaboration with general practices.

Our preliminary study was carried out in a group family medicine practice in Lausanne comprising seven GPs and four medical assistants, from February to April 2019. A research assistant provided potential participants with oral and written information about the study and obtained their written consent if they decided to participate. All patients over 18 years old who consulted one of the GPs during the study period were eligible to participate. However, patients who were unable to complete the questionnaire (e.g., because of dementia or blindness) were excluded.

### Data

Data were collected from patients, their medical records, and their physicians. Patients completed the IMSA questionnaire. which includes 20 questions about the biopsychosocial complexity of the patient’s clinical situation and the adequacy of their care. Complexity is divided into the biological, psychological, and social domains (with five questions each), and adequacy of care is rated within a fourth, a healthcare system domain (with five further questions). Each domain is sub-divided into the three separate time periods of assessment: past experience, current experience, and future prognosis. Each IMSA answer is rated from 0 (absence of complexity/problems of adequacy) to 3 (important complexity/inadequacy of care), resulting in a total possible IMSA score of between 0 and 60 and four domain scores of between 0 and 15. The questions on historical biological chronicity (Q1), current diagnostic/therapeutic challenges (Q4), and work and leisure activities (Q9) include two or three sub-questions whose scores are recoded into one pooled score using an algorithm (see Supplementary File for a typical grid of individual results) [23]. Patients completed the questionnaire using a tablet computer (HP Slate 7 VoiceTab Ultra 3900nf) in the waiting room either before or after (or before and after) their consultation. The median filling time was 8.1 min for a pre-consultation fill and 9.9 min for a post-consultation fill.

In addition to the IMSA data, the research assistant collected data from each patient’s medical record concerning sociodemographic (age and sex) and medical information (number of comorbidities and number of treatments prescribed for at least 3 months) and information about healthcare services used over the last 12 months (number of consultations, ER visits, and hospitalizations, plus total costs invoiced). The number of comorbidities only concerned diseases that were currently affecting the patient (past surgical problems or past diseases were not investigated). The number of treatments concerned medications prescribed by a general practitioner (GP) for a duration of 3 months or more. Finally, the research assistant asked GPs for their opinions on the potential benefits of each patient being assigned a case manager. The four possible answers were “*Yes, very useful”, “Yes, quite useful”, “No*, probably unnecessary *”*, and *“*unnecessary ”. The GPs were not informed about their patients’ IMSA scores.

### Statistical analysis

RedCap software [31] was used to create the questionnaire and build and manage the database. Overall IMSA scores and biological, psychological, social and healthcare system sub-dimension scores were calculated automatically as patients completed the questionnaire, as per the rules for IMSA calculating [23] (see above and Supplementary File). All ensuing statistical analyses used Stata software (v16).

Firstly, descriptive statistics about patients’ personal and health characteristics were calculated, as were the distribution of the IMSA (global scores and scores according to the four dimensions) and the distribution of GPs’ opinions regarding the benefits of introducing case management for each patient individually.

Secondly, we evaluated each IMSA item’s value as a predictor of GPs’ opinions about the benefits of assigning a case manager by using univariate and multivariate logistic models (using backwards stepwise selection and *p* values < 0.05). Patients’ personal (sex and age) and health data (number of diseases and treatments, total annual cost, annual numbers of GP consultations, ER visits, and hospitalizations) were also studied as predictive factors. GPs’ opinions on the utility of assigning each patient a case manager were dichotomized into “Yes” (including *“Yes, absolutely useful”* and *“Yes, quite useful”*) or ”No” (including “*No, not necessarily useful*” and “*Not useful at all*”).

Thirdly, predictive items selected from the final multivariate model were used to create an overall case management questionnaire score. The score’s sensitivity and specificity and the area under a ROC curve were used to define a threshold for selecting patients to be assigned a case management.

## Results

Of 534 eligible patients, 19% refused to participate because of a lack of time and 19% because of a lack of interest. The study participation rate was 62% (n = 331). Participants’ characteristics are described in Table 1. Median participant age was 54 years old, and most were women (60%). The mean numbers of ER visits and hospitalizations in the last 12 months were respectively 0.71 and 0.13. The median IMSA score was 8 (out of 60; range 0–33]; mean = 10). GPs reported that, in their opinion, about 40% of their patients might benefit from being assigned a case manager and that this would be very useful for 4% of them (Table 1).

**Table 1:** Sample characteristics (N = 331)

	Mean or %	Median
**Female sex**	40.2	
**Age**	54.3	54.27
**Number of current diseases**	4.1	3
**Number of current treatments**	2.6	2
**Number of GP consultations in last 12 months**	5.1	3
**Cost of GP consultations in last 12 months (Swiss francs)**	611	457
**Number of emergency room visits in last 12 months**	0.71	0
**Number of hospitalizations in last 12 months**	0.13	0
**Total IMSA questionnaire score (/60)**	10.27	8
**IMSA biological score (/15)**	4.23	4
**IMSA psychological score (/15)**	3.18	2
**IMSA social score (/15)**	1.63	1
**IMSA healthcare score (/15)**	1.23	1
Absolutely useful	4.3	
Quite useful	36.6	
Not necessarily useful	21.6	
Not useful at all	37.5	

**Fig. 1:**
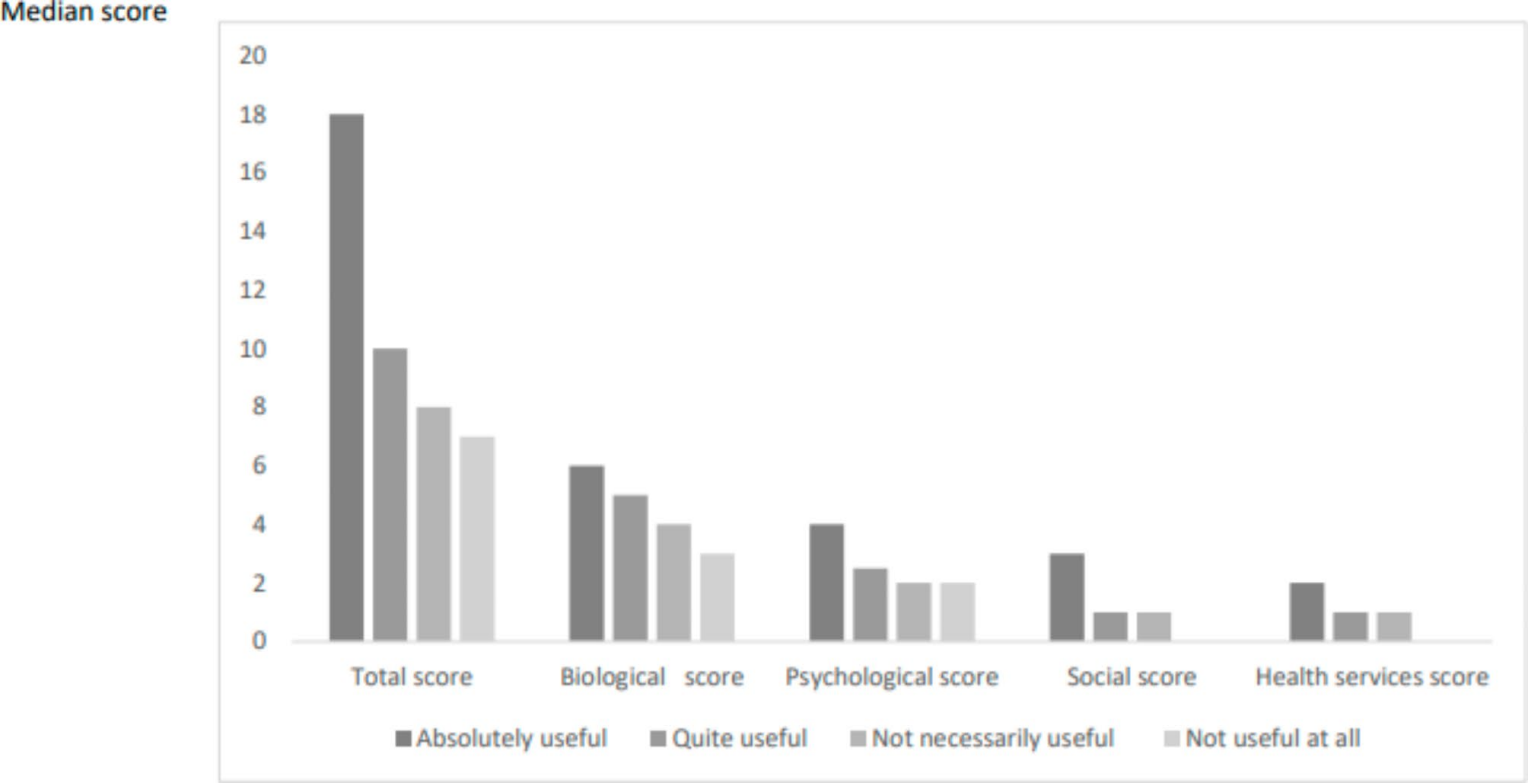
Distribution of the IMSA questionnaire scores (total and by sub-dimensions) in relation to GPs’ opinions about the potential benefits of assigning patients a case manager

The median IMSA score was higher among patients whom GPs thought would probably benefit from case management (absolutely useful or quite useful): 18/60 points when case management was judged “*absolutely useful*”, 10/60 when “*quite useful*”, 8/60 when “*not necessarily useful*”, and 7/60 when “*not useful at all*”. The same trend was observed for each IMSA sub-dimension score (Fig. 1).

Table 2 shows the statistical predictive role of each IMSA item regarding the GPs’ opinions about the benefits of patients having case managers. In univariate analyses, 11 of the 20 IMSA items were predictive of GPs’ opinions about case management. Indeed, all the other variables (age, sex, number of GP consultations, number of treatments, number of diseases, and ER visits, plus total costs over the last 12 months) were individually predictive too, except for the number of hospitalizations. However, our final, multivariate logistic regression model retained only three predictive items from the original IMSA questionnaire (with odds ratio > 1, *p*-value ≥ 0.05), and no other variables reached a level of statistical predictive significance when added to them. The first predictive item—including the two sub-questions (Q1a and Q1b) of “*Did you experience any physical problem in the past 5 years*?” and “*Do you suffer from one or more long-lasting or chronic diseases (such as diabetes, high blood pressure, rheumatoid arthritis, lung disease, or cancer)?*”—was related to the patient’s medical history (OR = 2.11, 95%CI 1.46–3.04). The second predictive item (Q3), dealing with quality of life, was “*How much were your daily activities (such as job, house-keeping, hobbies, going out…) restricted by physical problems during last week*?” (OR = 1.55, 95%CI 1.23–1.96). The third predictive item (Q9a, Q9b, Q9c) asked, “*Do you have a job?”, “If no specify [your employment status]”*, and *“Have you got activities in your spare time such as volunteering, courses, sports, clubs…?”*) addressing the issue of social support (OR = 1.86, 95%CI 1.48–2.35) (Table 2).

**Table 2:** Predictive value of the patient’s IMSA item responses for GPs’ opinions about the potential benefits of assigning the patient a case manager (logistic models)

	Single-variate models			Multi-variate models ¥			Multi-variate models ¥			Final multi-variate models £		
**Item**	**OR**	**95% [CI]**	**OR**		**95% [CI]**	**OR**		**95% [CI]**	**OR**		**95% [CI]**	
Q1	2.52	[1.79–3.56]	2.24		[1.52–3.29]				2.11		[1.46–3.04]	
Q2	1.43	[1.12–1.84]	1.04		[0.74–1.47]							
Q3	1.78	[1.43–2.21]	1.39		[1.02–1.90]				1.55		[1.23–1.96]	
Q4	1.98	[1.34–1.92]	0.99		[0.59–1.66]							
Q5	1.52	[1.20–1.93]	1.26		[0.86–1.85]							
Q6	1.17	[0.94–1.46]	1.13		[0.79–1.60]							
Q7	1.36	[1.05–1.77]	1.08		[0.75–1.53]							
Q8	1.13	[0.89–1.43]	0.57		[0.36–0.90]							
Q9	1.99	[1.61–2.47]	1.79		[1.39–2.30]				1.86		[1.48–2.35]	
Q10	1.51	[0.96–2.39]	113		[0.61–2.12]							
Q11	2.21	[1.49–3.30]	1.41		[0.83–2.42]							
Q12	1.71	[1.18–2.47]	1.23		[0.74–2.03]							
Q13	1.10	[0.62–1.97]	0.87		[0.44–1.73]							
Q14	1.49	[1.06–2.09]	0.92		[0.60–1.41]							
Q15	0.57	[0.17–1.85]	0.49		[0.13–1.85]							
Q16	1.36	[0.80–2.32]	0.97		[0.51–1.87]							
Q17	2.11	[1.23–3.61]	1.11		[0.61–2.05]							
Q18	1.54	[0.86–2.77]	1.66		[0.68–4.06]							
Q19	1.28	[0.97–1.68]	1.25		[0.85–1.8]							
Q20	1.30	[0.81–2.10]	0.73		[0.38–1.40]							
Age	1.04	[1.02–1.05]				1.02		[1.00–1.04]				
Sex	0.60	[0.39–0.96]				0.65		[0.37–1.14]				
Visit to GP*	1.14	[1.07–2.21]				1.05		[0.98–1.13]				
Diseases**	1.16	[1.08–1.24]				1.03		[0.93–1.13]				
Treatments**	1.34	[1.22–1.47]				1.18		[1.05–1.32]				
Cost*	1.00	[1.00–1.00]				1.00		[1.00–1.00]				
Visit to ER*	1.27	[1.05–1.54]				1.23		[0.99–1.52]				
Hospitalization*	1.53	[0.90–2.61]				1.03		[0.56–1.88]				

* last 12 monts, number of /** Number of current diseases or treatments

¥ Sociodemographic and health-related variables were tested independently in a model including all the IMSA questionnaire items

£ Forward stepwise regression

**Table 3:** Sensitivity and specificity of the short-form tool according to the threshold (real values)

	Percent of the sample	Sensitivity	Specificity
Score ≥ 1	88.4	97.0	17.5
Score ≥ 2	76.2	91.8	34.5
Score ≥ 3	61.0	83.5	54.6
Score ≥ 4	44.5	70.1	73.2
Score ≥ 5	48.5	48.5	87.1
Score ≥ 6	32.8	32.8	94.3
Score ≥ 7	4.9	11.2	99.5
Score ≥ 8	0.3	0.75	100

Short-form tool score = Q1 + Q3 + Q9

**Fig. 2:**
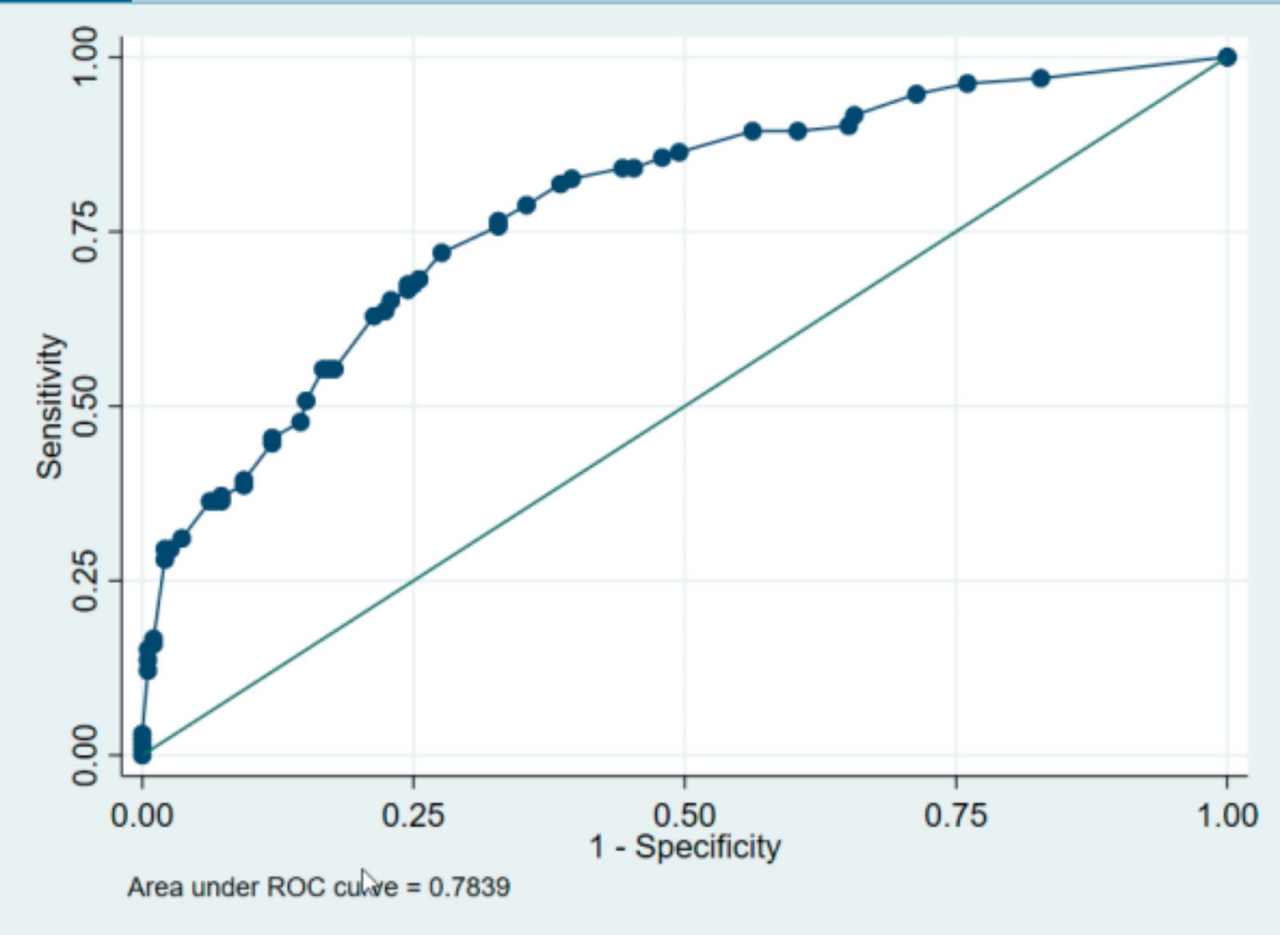
ROC curve obtained using the predictive items from the IMSA questionnaire as selected using our final multivariate logistic regression model, and the predicted values of the area under the curve

Using these three items, we built a new score based upon the same rules defined for the complete IMSA score (see above and Supplementary File). The area under the ROC curve was 78%. Table 3 shows the distribution of specificity and sensitivity according to the selected cut-off and the proportion of the population concerned. A cut-off at 4 gave a sensitivity of 70% and specificity of 73% and concerned about one patient in two to be eligible for case management. As our goal for the next part of the project was to use this short form as a pre-test before the administration of the complete IMSA, we chose to retain this threshold. (Table 3; Fig. 2: ROC curve, area under the curve including Q1 + Q3 + Q9 / score range = 0–9).

## Discussion

Our results showed that three items from the IMSA questionnaire were sufficient to predict GPs’ opinions about whether their patients could be expected to benefit if assigned a case manager. Those items addressed the patient’s physical health (existing chronic diseases), quality of life in relation to existing diseases, and their social situation (Table 4).

**Table 4:** Three items from the IMSA questionnaire retained to predict the potential utility of assigning a case manager

IMSA questions	Answer	Score calculation
Q1. 1a. Did you experience any physical problems in the past 5 years? 1b. Do you suffer from one or more long-lasting or chronic diseases?	|a|0 No, I did not |b|0 Yes, I experienced physical problems but for a period shorter than 3 months |c|1 Yes, Iexperienced physical problems for a period longer than 3 months |d|1 Yes, in the last 5 years I have experienced several short periods with physical problems |a|0 I don’t have any long-lasting or chronic diseases |b|2 I suffer from one long-lasting or chronic disease |c|3 I suffer from several long-lasting or chronic diseases	if 1a) a or 1a) b and 1b) a, = 0 if 1a) c or 1a) d, = 1 if 1b) b, = 2 if 1b) c, = 3
Q3. How much were your daily activities (such as job, house-keeping, hobbies, going out…) restricted by physical problems during the last week?	|a|0 I have no, or insignificant, physical problems |b|1 My daily activities are not or are only mildly influenced by the physical problems that I experience |c|2 My daily activities are moderately influenced by physical problems |d|3 My daily activities are severely influenced by physical problems	
Q9. 9a. Do you have a job? 9b. if not, please specify your employment status. 9c. Have you got activities in your spare time such as volunteering, courses, sports, clubs…?	|a| Yes |b| No |a| I am a student |b| I am retired |c| I am a housewife taking care for the household and others |d| I am disabled |e| I have been on sick leave for more than 6 months |a| Yes |b| No	if 9a)a and 9c)a, then = 0 if 9a)a and 9c)b, then = 1 if 9a)b and 9c)a, then = 2 if 9a)b and 9c)b, then = 3

GPs’opinions: Absolutely useful (N = 14); Quite useful (N = 120); Not necessarily useful (N = 71); Not useful at all (N = 123)

IMSA Scores: Total (median / 60); Biological score (median / 15); Psychological score (median / 15); Social score (median / 15); Healthcare services score (median / 15)

These items involve very simple questions and easily collectable answers. In most cases, the information may well already exist in patients’ medical records, and a score could be calculated automatically. The fact that no additional data collection is necessary should be a key argument for proposing the use of this approach in general practices. Indeed, time is often a significant and costly issue for patients consulting a GP, the GPs themselves, and their administrative employees. Using a short questionnaire to collect a very limited amount of data might be appropriate in terms of acceptability by patients. Finding from another part of the present study [32] and the study by de Oliveira conducted in primary care facilities in Brazil [27], the only factor limiting study participation was the time necessary to complete the questionnaire. This limitation would be minimized by using three questions. Another advantage of this tool is that it could be used to characterize and stratify all the patients in practices or clinics. Different thresholds could be used to identify patients with a very high, intermediate, or low need for case management. Upstream use of the tool could have two interesting consequences: firstly, targeted collective interventions could be proposed to patients, and secondly, a primary care facility could adjust its resource allocation system depending on its population’s needs. Further research should be conducted to explore these possibilities.

Our results underline the importance of the dimensions of the patient’s social environment and quality of life when considering the management of their chronic diseases. Garcia et al. also mentioned the relevance of social support (in addition with patient ‘s motivation) in their research on patients who might benefit from care management [20]. In the context of frequent users of ERs, Hudon et al. also highlighted the importance of social factors and quality of life. Their case-finding tool was developed and validated to identify patients with complex needs in ERs and, among its six dimensions, it includes social support and limitations due to pain [21]. All these results underlined the necessity to reinforce holistic approaches to patient health management, especially for those with complex needs, as advocated by Engel’s biopsychosocial model [33], and primary care is probably the most appropriate setting in which to integrate such approaches.

The INTERMED approach was developed and validated in the hospital setting, with patients cared for by specialists. Complexity in general practice has different contextual aspects to complexity in a hospital context. Our study raises issues including, how to define complexity, and what GPs seek to gain from case management. Firstly, complexity is a multidimensional concept that is difficult to grasp and probably also depends significantly on context [34]. Indeed, another part of our research program investigated GPs’ definitions of complexity. This qualitative study showed that GPs’ representations of complexity were very broad and heterogeneous, which could affect the use of a tool like the INTERMED (Donnet, manuscript in preparation). Furthermore, this study highlights that some of the dimensions of complexity, as they are perceived by GPs, are not identified in the IMSA (such as difficult or unreliable patients, case-provider fatigue or conflict). Secondly, there are many other elements—besides using a tool that contributes to finding appropriate indications for case management—that can influence a referral to or proper use of a case manager. The reasons why GPs might feel it is advantageous to work with a case manager are probably diverse, partly because this role does not yet exist within Switzerland’s general practices. Informal discussions with GPs revealed that some GPs would be happy to work with case managers, mainly to help them deal with “difficult” patients. However, it is questionable whether relieving GPs of the burden of their most demanding patients is truly within the scope of a case manager.

In a recent literature review we conducted about intervention that could improve coordination in PC settings, we reported that interventions involving “case managers” were associated with the greatest number of articles describing positive effects [34]. These results have been reinforced through another recent review about nursing care coordination for patients with complex needs in PC [14]. Many countries such as Australia, New Zealand, Sweden, the United Kingdom and the Netherlands, have long developed the role of CM within primary care settings. The main mission of CM is to coordinate care of patients having one or more pathologies but the tasks can vary from overseeing patient parameters to develop patient empowerment. The positive effects include not only patients clinical outcomes but also patients and professionals satisfaction. Finally, this management can lead to a decrease in the length of hospital stays and rehospitalizations [35, 36].

## Limitations

Because a significant proportion of eligible patients (38%) refused to participate, due to a lack of time or interest, the sample could have been biased. However, the direction of that bias is difficult to estimate. Data collected from patients’ medical records were not standardized. Indeed, medical data were based on GPs’ records of diseases and treatments. Data on the use of care is more reliable because some of it is automatically accounted for in the medical practice’s software. The present study’s results were only based on the opinions of seven GPs who all worked in the same group family medicine practice. Their points of view about complexity and the role of case managers may not be representative. For instance, a practice’s rural or urban location or how it is organized could play a role in GPs’ opinions about case management, as could GPs’ age or gender. The fact that the dimensions highlighted in this study are common to other research is reassuring, however. We nevertheless recommend a validation study on another general practice dataset.

### Implications for practice

Based on the results of this study, the three IMSA items (Table 4), identified as predictors of a GP perceiving a potential benefit from case management, could be used as a rapid, simple pre-screening tool in general practices. It could easily be proposed to all patients in primary care. In a second step, the entire IMSA could be used, but within a team discussion, also including the patient to identify specific domains to work on.

## Conclusion

Identifying patients who may benefit from case management remains a challenge for primary care professionals. Beyond the use of one or more specific tools to assist in identifying the relevant patients, a primary care team’s experience and knowledge of each patient’s uniqueness will also play a role. The final decision should depend on the objectives and expectations of all the stakeholders. Finally, the organizational structure of the particular healthcare system and services will influence options for decision-making (e.g., home-based care for highly complex patients).

## Electronic supplementary material

Below is the link to the electronic supplementary material.

Supplementary Material 1 - The IMSA questionnaire.

## Data Availability

Data could be available from the corresponding author on reasonable request.

## References

[1] Fraher EP (2020). Primary Care Teams: Past, Present and Future. J Am Board Fam Med.

[2] Kringos DS, Policies (2015). Building primary care in a changing Europe.

[3] Linzer M, Poplau S (2017). Building a Sustainable Primary Care Workforce: Where Do We Go from Here?. J Am Board Fam Med.

[4] Ballard T (2013). What sustainability means for primary care: primary care leads to better overall resource use and higher quality outcomes. Br J Gen Pract.

[5] Bodenheimer T (2014). The 10 building blocks of high-performing primary care. Ann Fam Med.

[6] Pandhi N (2018). Developing primary care teams prepared to improve quality: a mixed-methods evaluation and lessons learned from implementing a microsystems approach. BMC Health Serv Res.

[7] Fraher EP (2020). Primary Care Teams: Past, Present and Future. J Am Board Family Med.

[8] Morgan S, Pullon S, McKinlay E (2015). Observation of interprofessional collaborative practice in primary care teams: An integrative literature review. Int J Nurs Stud.

[9] Wranik WD, et al., *Implications of interprofessional primary care team characteristics for health services and patient health outcomes: A systematic review with narrative synthesis*. Health Policy, 2019. 10.1016/j.healthpol.2019.03.015 30955711

[10] Teper MH (2020). Understanding Barriers to and Facilitators of Case Management in Primary Care: A Systematic Review and Thematic Synthesis. The Annals of Family Medicine.

[11] Challis D (2011). Implementation of case management in long-term conditions in England: survey and case studies. J Health Serv Res Policy.

[12] Joo JY, Huber DL (2018). Scoping Review of Nursing Case Management in the United States. Clin Nurs Res.

[13] Stokes J (2015). Effectiveness of Case Management for ‘At Risk’ Patients in Primary Care: A Systematic Review and Meta-Analysis. PLoS ONE.

[14] Karam M (2021). Nursing Care Coordination for Patients with Complex Needs in Primary Healthcare: A Scoping Review. Int J Integr Care.

[15] Bujold M (2017). Decisional needs assessment of patients with complex care needs in primary care: a participatory systematic mixed studies review protocol. BMJ Open.

[16] Askerud A, Conder J (2017). Patients’ experiences of nurse case management in primary care: a meta-synthesis. Aust J Prim Health.

[17] Gravelle H (2007). Impact of case management (Evercare) on frail elderly patients: controlled before and after analysis of quantitative outcome data. BMJ.

[18] Reilly S (2011). Case management for people with long-term conditions: impact upon emergency admissions and associated length of stay. Prim Health Care Res Dev.

[19] Hudon C (2018). Case management in primary care for frequent users of healthcare services with chronic diseases and complex care needs: an implementation and realist evaluation protocol. BMJ Open.

[20] Garcia ME (2018). Which Complex Patients Should Be Referred for Intensive Care Management? A Mixed-Methods Analysis. J Gen Intern Med.

[21] Hudon C (2021). CONECT-6: a case-finding tool to identify patients with complex health needs. BMC Health Serv Res.

[22] Marcoux V (2017). Screening tools to identify patients with complex health needs at risk of high use of health care services: A scoping review. PLoS ONE.

[23] van Reedt Dortland AKB (2017). Assessment of Biopsychosocial Complexity and Health Care Needs: Measurement Properties of the INTERMED Self-Assessment Version. Psychosom Med.

[24] Huyse FJ (1999). "INTERMED”: a method to assess health service needs. I. Development and reliability. Gen Hosp Psychiatry.

[25] Stiefel FC (1999). "INTERMED”: a method to assess health service needs. II. Results on its validity and clinical use. Gen Hosp Psychiatry.

[26] Bleijenberg N (2014). Associations between Frailty, Complex Care Needs and Quality of Life in Multi-Morbid Older People. J Frailty Aging.

[27] de Oliveira CA, et al., *Health Complexity Assessment in Primary Care: a validity and feasibility study of the INTERMED tool* medRxiv, 2020: p. 2020.10.21.20216929. 10.1371/journal.pone.0263702 PMC8856552 35180262

[28] Kathol RG, et al., *Creating clinical and economic “wins” through integrated case management: lessons for physicians and health system administrators* Prof Case Manag, 2011. 16(6): p. 290-8; quiz 299–300. 10.1097/NCM.0b013e318230ea5b 21986971

[29] Schutz Leuthold M (2020). Protocol for an implementation and realist evaluation of a new organisational model for primary care practices in the canton of Vaud, Switzerland. BMJ Open.

[30] Cohidon C, Cornuz J, Senn N (2015). Primary care in Switzerland: evolution of physicians’ profile and activities in twenty years (1993–2012). BMC Fam Pract.

[31] Harris PA (2009). Research electronic data capture (REDCap)—A metadata-driven methodology and workflow process for providing translational research informatics support. J Biomed Inform.

[32] Leckwyck LV, Gallay E, Bourquin C, Stiefel F, Cohidon C, Senn N. Bio–Psycho–Social Needs Assessment in Family Medicine:Acceptability of the Intermed Self–Assessment]. Praxis (Bern 1994). 2022;111(3):135–140. 10.1024/1661-8157/a003818 35232262

[33] Engel GL (1980). The clinical application of the biopsychosocial model. Am J Psychiatry.

[34] de Jonge P, Huyse FJ, Stiefel FC (2006). Case and care complexity in the medically ill. Med Clin North Am.

[35] Cardinaux R, et al., *Interventions to improve care coordination in primary care: A narrative review*. J Prim Care Gen Pract, 2020. 4(1).

[36] Doty MM, Fryer AK, Audet AM (2012). The role of care coordinators in improving care coordination: the patient’s perspective. Arch Intern Med.

