# Supplementary material for: Identifying complex patients in family medicine for potential benefit from a case manager: a short questionnaire derived from the INTERMED Self-Assessment (IMSA) questionnaire
Source: BMC Prim Care. 2022 Nov 4;23:276. doi: 10.1186/s12875-022-01876-8 (PMC9636696; doi:10.1186/s12875-022-01876-8)
Supplement: Supplementary file 1 — Supplementary Material 1 - The IMSA questionnaire. [file 12875_2022_1876_MOESM1_ESM.pdf]

Supplementary file. The IMSA questionnaire

PRE - 1 What is your level of understanding of the language of the country you live in?
|a| Native speaker
|b| Good knowledge of language
|c| Moderate knowledge of language
|d| Poor knowledge of language

PRE - 2 Who fills in the questionnaire?
|a| I will fill in the questionnaire myself
|b| I will fill in the questionnaire myself though aided by someone else
    |a| Partner/Family member/Friend
    |b| Nurse
    |c| Researcher
    |d| Other (please specify) ______________________________
|c| Someone else will fill in the questionnaire after consulting me
    |a| Partner/Family member/Friend
    |b| Nurse
    |c| Researcher
    |d| Other (please specify) ______________________________

Add 1 - Are you taking medications almost every day?
|a| I don't take any regular medication
|b| I do take one regular medication
|c| I do take several medications

If so, how many different medications each day you take? __________________

Historical - Biological chronicity

1a Did you experience any physical problem in the past 5 years?
|a|**0** No, I did not
|b|**0** Yes, I experienced physical problems but for a period shorter than 3 months
|c|**1** Yes, I did experience physical problems for a period longer than 3 months
|d|**1** Yes, in the past 5 years I have experienced several short periods with physical problems

1b Do you suffer from one or more long-lasting or chronic diseases (such as diabetes, high blood pressure, rheumatoid arthritis, lung disease or cancer)
|a|**0** I don't have a long-lasting or chronic disease
|b|**2** I suffer one long-lasting or chronic disease
|c|**3** I suffer several long-lasting or chronic diseases

To be filled in by your health professional

If 1a) a or 1a) b and 1b) a, score on historical biological chronicity = **0**
If 1a) c or 1a) d, score on historical biological chronicity = **1**
If 1b) b, score on historical biological chronicity = **2**
If 1b) c, score on historical biological chronicity = **3**

Historical -Diagnostic dilemma
2 How difficult has it been in the past 5 years to diagnose the physical problems you experienced?

|a|0 I did not suffer of any physical problem in the past 5 years
|b|0 The reason for my problems was immediately clear
|c|1 After some routine investigations the reason for my problems was identified
|d|2 After a lot of investigations the reason for my problems was identified
|e|3 Even though a series of investigations have been taken into effect, the origins of my problems were never diagnosed

Current - Symptom severity
3 How much were your daily activities (such as job, house-keeping, hobbies, going out…) restricted by physical problems during the last week?
|a|0 I have no, or insignificant, physical problems
|b|1 My daily activities are not or are only mildly influenced by the physical problems that I experience
|c|2 My daily activities are moderately influenced by physical problems
|d|3 My daily activities are severely influenced by physical problems

Current – Diagnostic/therapeutic challenge
4a Do you think your doctors understand the origin of your current physical problem/s?
|a|0 I do not have physical problems at present
|b|1 My doctors do understand the origin of my current physical problem/s
|c|2 My doctors understand the origin of my current physical problem/s but they have some doubts
|d|3 My doctors have many doubts about the origin of my current physical problem/s
|e|3 My doctors still have to find the origin of my current physical problem/s

4b Do you think you are receiving the appropriate treatment for your current physical problem/s?
|a|0 I do not have physical problems at present
|b|1 I am receiving the appropriate treatment for my current physical problem/s
|c|2 I have some doubts about the appropriateness of the treatment for my current physical problem/s
|d|3 I have many doubts about the appropriateness of the treatment for my current physical problem/s
|e|3 The appropriate treatment for my current physical problem/s is still to be found

To be filled in by your health professional
If 4a) and 4b) are both scored a, score on current diagnostic/therapeutic challenge = **0**
If 4a) or 4b) is scored b, score on current diagnostic/therapeutic challenge = **1**
If 4a) or 4b) is scored c, score on current diagnostic/therapeutic challenge = **2**
If 4a) or 4b) is scored d or e, score on current diagnostic/therapeutic challenge = **3**

Historical – Coping
5 In the past 5 years, how did you cope with stressful, difficult situations?
|a|0 Generally speaking, I have always been able to cope with stressful, difficult situations
|b|1 Sometimes I had difficulties in coping with stressful, difficult situations, which sometimes resulted in tensions and problems with my partner, family or other people.
|c|2 I often experienced difficulties with stressful, difficult situations, which often led to tensions and problems with my partner, family or other people
|d|3 I always experience difficulties with stressful, difficult situations. They upset me and make me tense

Historical – Mental health
6 In your past, have you ever had psychological problems, such as being tense, anxious, down/blue or confused?
|a|0 No, almost never
|b|1 Yes, however without clear influence on my daily life
|c|2 Yes and it influenced my daily life
|d|3 Yes and these problems have had or still have a long-lasting effect on my daily life

Current – Resistance to treatment
7 Do you think it is difficult to follow your health caregivers' recommendations?
|a|0 No, I don't think this is difficult
|b|1 Yes, I think this is difficult, but I manage

|c|2 Yes, I think this is difficult, sometimes I manage, sometimes I don’t
|d|3 Yes, I think this is too difficult, most of the times I don’t manage

Current – Mental health symptoms
8 At present, are you experiencing psychological problems, such as being tense, anxious, down/blue or confused?
|a|0 No, no problems
|b|1 Yes, mild problems that do not affect my ability to do daily activities
|c|2 Yes, moderate problems that affect my ability to do daily activities a little
|d|3 Yes, severe problems that affect my ability to do daily activities a lot

Historical – Job and leisure
9a Do you have a job?
|a| Yes
|b| No

9b If you said No, please specify:
|a| I am a student
|b| I am retired
|c| I am a housewife taking care for the household and others
|d| I am disabled
|e| I am more than 6-months on sick leave

9c Have you got activities in your spare time such as volunteering, courses, sports, clubs…?
|a| Yes
|b| No

To be filled in by your health professional
If 9a) a and 9c) a, score on current job and leisure = **0**
If 9a) a and 9c) b, score on current job and leisure = **1**
If 9a) b and 9c) a, score on current job and leisure = **2**
If 9a) b and 9c) b, score on current job and leisure = **3**

Historical – Social relationships
10 How do you generally relate to other people?
|a|0 I have a sufficient amount of contacts with others and socialize well
|b|1 I have contacts with others, though every now and then it might become tense
|c|2 It is difficult for me to initiate or maintain contacts or friendships with others
|d|3 Contacts or friendships often deteriorate into quarrels and conflicts

Current – Residential stability
11 Is your home living situation satisfactory? Or are adjustments needed, such as home modifications, receiving home care, or going to live somewhere else?
|a|0 No adjustments needed, I can manage my home situation
|b|1 No adjustments needed, as there is enough support and care by others
|c|2 Adjustments are needed, however not immediately
|d|3 Immediate adjustments are needed

Current – Social support
12 Is assistance from your partner, family, colleagues or friends available for you at any time?
|a|0 I am not in need of assistance
|b|0 Yes, assistance is available at all time
|c|1 Yes, assistance is available but not at all times
|d|2 The assistance I get is very limited
|e|3 No assistance is available

Historical – Access to care

13 Do you experience problems in getting the care you need due to living too far away, or not having any insurance, or not speaking the language very well, or differences in culture?
|a|0 No, these are not problems for me
|b|1 Yes, I experience some of these problems every now and then
|c|2 Yes, I often experience some of these problems
|d|3 Yes, some of these are big problems for me

Historical – Treatment experience
14 How did you experience your contacts with doctors and healthcare providers in the last 5 years?
|a|0 I never had problems with doctors and healthcare providers
|b|1 I or someone close to me had negative experience(s) with doctors and healthcare providers
|c|2 I have changed doctors and healthcare providers as a result of a negative experience
|d|3 I frequently have changed doctors and healthcare providers because of negative experiences or lack of trust

Current – Organization of care
15 Who are the healthcare providers who take care for you at the moment? [multiple answers allowed]
|a| I don't receive any care
|b| Primary care physician/general practitioner
|c| One medical specialist (such as: respiratory physician, cardiologist, surgeon, general physician) for physical problems
|d| Several medical specialists for physical problems
|e| One or more specialists for mental problems (such as: psychiatrist, psychologist, specialist for substance abuse…)
|f| Social worker
|g| Home nurse
|h| I'm currently admitted to a hospital
|i| I'm currently admitted to a psychiatric hospital
|j| Other (please specify) ___________________________________

To be filled in by your health professional

If 15) a or b or f or g, score on current organization of care = **0**
If 15) c or d or e, score on current organization of care = **1**
If 15) c or d and e, score on current organization of care = **2**
If 15) c or d and e plus h or i, score on current organization of care = **3**

Current – Coordination of care
16 To what extent do your doctors and healthcare providers work together?
|a|0 I do not receive care or my care is provided by just one doctor
|b|0 My doctors and healthcare providers work together well
|c|1 My doctors and healthcare providers work together, however sometimes more communication is needed
|d|2 My doctors and healthcare providers do not work together quite well, leading to problems every now and then
|e|3 My doctors and healthcare providers do not work together

Prognosis – Complications and threat
17 In the next 6 months, do you expect your physical health to change? [Try to make the best estimate]
|a|0 In the next 6 months I expect my physical health to remain the same or get better
|b|1 In the next 6 months I expect only a slight worsening of my physical health
|c|2 In the next 6 months I expect a worsening of my physical health
|d|3 In the next 6 months I expect a considerable worsening of my physical health

Prognosis – Mental health threat
18 In the next 6 months, do you expect your psychological well being to change? [Try to make the best estimate]
|a| 0 In the next 6 months I expect my psychological well-being to remain the same or get better
|b|1 In the next 6 months I expect only a slight worsening of my psychological well-being

|c|2 In the next 6 months I expect a worsening of my psychological well-being
|d|3 In the next 6 months I expect a considerable worsening of my psychological wellbeing

Prognosis – Social vulnerability
19 In the next 6 months do you expect that a change will be needed in the way you are currently living? [Try to make the best estimate]
|a|0 In the next 6 months there is no need to change the way I am currently living
|b|1 In the next 6 months I am able to stay or return to my current living situation. However homecare is required
|c|2 In the next 6 months a change to another living situation will be needed
|d|3 A change to another living situation is needed immediately

Prognosis – Health system impediments
20 In the next 6 months, do you expect that you will be in need of more help and support? [Try to make the best estimate]
|a|0 I expect in the next 6 months that my need of care will remain the same or become less
|b|1 I expect in the next 6 months that my need of care will increase
|c|2 I expect in the next 6 months that my need of care will increase very much
|d|3 I expect in the next 6 months that my need of care will increase very much and additional services will be necessary
